# Four years stability of type D personality in patients with moderate to severe psoriasis and its implications for psychological impairment^☆^^☆☆^

**DOI:** 10.1016/j.abd.2021.02.005

**Published:** 2021-07-15

**Authors:** Paula Aguayo-Carreras, José Carlos Ruiz-Carrascosa, Ricardo Ruiz-Villaverde, Alejandro Molina-Leyva

**Affiliations:** a Department of Dermatology, Hospital San Cecilio, Granada, Spain; b Department of Dermatology, Hospital Universitario Virgen de las Nieves, Granada, Spain

**Keywords:** Anxiety, Depression, Psoriasis, Type D personality

## Abstract

**Background:**

Psoriasis is a systemic auto-inflammatory disease that is related to an increased risk of organic and psychological comorbidities. Type D is a stable personality trait in healthy subjects but there is no data regarding its stability in patients with moderate-severe psoriasis.

**Objectives:**

To assess the stability of type D personality in patients with moderate to severe psoriasis as well as assessing the influence of type D personality on anxiety and depression.

**Methods:**

Prospective cohort study. Forty psoriasis patients with type D personality and sixty-six patients with psoriasis without type D personality were included in the study. Participants completed the DS14 test and HADS at baseline and four years later.

**Results:**

At baseline, the prevalence of type D personality was 37.7% and at week 208 it was 27.3%. The stability of type D personality was higher in patients with an incomplete education level and in those who were separated/divorced or windowed. During follow-up, 15% of patients developed type D personality. Male sex, having topical treatment, the presence of previous depression, anxiety, and high levels of negative affectivity at baseline increase the risk of developing type D personality.

**Study limitations:**

Sample size, psoriasis severity restricted to moderate and severe and all patients being under treatment for psoriasis.

**Conclusions:**

The presence of type D personality varies over time in psoriasis patients. Therefore, type D personality is possibly more a state than a trait phenomenon, modified by environmental factors. Type D personality is associated with a higher risk of anxiety.

## Introduction

Negative Affectivity (NA) and Social Inhibition (SI) are the traits that characterize type D's “distressed” personality. The person is unable to gain adequate social support, in part due to the high negative affectivity they experience.^1^

The prevalence of type D personality in healthy subjects in several studies ranges between 20% and 24.1%.^2^, ^3^ According to recent research, the frequency of type D personality is increased in patients with psoriasis in contrast to the general population, in the range of 25%–38.7%, and in psoriatic patients, there is also a higher prevalence of anxiety and depression.^4^, ^5^, ^6^ The psychological damage that accumulates over the years in a chronic disease such as psoriasis could be related, at least in part, to a higher prevalence of type D personality in patients with psoriasis.^7^

Since these are developmentally dynamic constructs, personality traits are subject to change but also to stability throughout life.^8^ According to Kupper et al. the stability of type D personality over time is determined by genetic and environmental factors.^9^ A study of Loosman et al. maintains that type D personality is variable over time and its stability is related to the stability of anxiety and depressive symptoms.^10^ They suggest that type D personality could be more of a state than a characteristic phenomenon.

There is no data on Type D stability in patients with moderate to severe psoriasis who may experience changes in the presence or absence of disease over time. Conden et al. shows that the prevalence of type D personality was 25.1% one month after myocardial infarction and regarding stability, 6.1% of the patients maintained type D personality during hospitalization and one month and twelve months later.^11^ Martens et al. show that the prevalence of type D personality was 18.3% during hospitalization for myocardial infarction, and 23.2% at eighteen months post-myocardial infarction.^12^ However, studies have shown contradictory results; type D characterization changed before and after cardiac surgery in about 60% of patients.^13^ In contrast to acute events such as myocardial infarction or cardiac surgery, psoriasis is a chronic disease and therefore the stability of type D personality could be higher with the consequences that this entails.

The aim of this study is to determine (1) the stability of type D personality over a four-year period in patients with moderate to severe psoriasis; (2) the factors associated with its stability; (3) the association of these personality traits with depression and anxiety (4) and the factors associated with new-onset of type D personality and the influence of type D personality on anxiety and depression.

## Methods

### Design and study population

Prospective cohort study. The study sample was consecutively recruited among patients with moderate-severe psoriasis who attended their scheduled follow-up visits at the Psoriasis Unit of Hospital Universitario San Cecilio. The Psoriasis unit serves all patients with moderate to severe psoriasis in our dermatology clinic. All participants provided informed consent to participate in the study. The ethics committee of Hospital Universitario San Cecilio approved the study. (REF:1505-N-19).

The authors’ sample in the study is a representative sample of patients with psoriasis that can be found in a dermatology consultation since it has been extracted from the psoriasis unit that attends all patients with moderate to severe psoriasis in the dermatology clinic with a reference population of 450,000 inhabitants.

A questionnaire with DS14 and HADS tests was provided at baseline and at week 208.

### Inclusion and exclusion criteria

The inclusion criteria were as follows: diagnosis of moderate-severe cutaneous psoriasis, aged 18 or older and granted informed consent.

The severity of psoriasis was assessed according to the consensus document of the Spanish Academy of dermatology.^14^

“Moderate to severe psoriasis is that which requires or has previously required systemic treatment, this includes conventional drugs, biological agents or photochemotherapy. Systemic treatment is indicated in patients with psoriasis in the following situations: (a) disease not controlled with topical treatments; (b) extensive disease (BSA > 5%–10%) (c) PASI > 7–15; (d) rapid worsening; (e) involving visible areas; (f) functional impairment (palmoplantar, nail, genital or scalp involvement); (g) subjective perception of severity (DLQI > 6–10); (h) extensive erythroderma or pustular psoriasis; and (i) disease associated with psoriatic joint disease”.

### Exclusion criteria

Are summarized in the following: refusal to participate in the study, active dermatological diseases other than psoriasis, treatment with psychoactive drugs, intellectual disability, active malignant disease, and alcohol abuse.

Through clinical interviews and physical examinations, all biometric parameters and sociodemographic data were collected. Arterial hypertension, dyslipidemia and diabetes mellitus were three physical comorbidities that were considered for the analysis as long as the patient was receiving active treatment. The severity of psoriasis was assessed by a trained dermatologist by clinical interview, physical examination, review of medical records, calculation of BSA affected by Psoriasis and Severity Index and Psoriasis Area (PASI).^15^

### Main endpoints of interest and sources of information

The authors’ main variables of interest were the presence of type D personality assessed by the questionnaire DS14, and the presence of anxiety and depression, which was measured by the Hospital Anxiety and Depression Scale (HADS).

The DS14 questionnaire, validated in the Spanish population, is widely used to assess the presence of type D personality.^1^ It is made up of 14 items, 7 related to negative affectivity and 7 to social inhibition. Each is scored from 0 to 4; and a score equal to or greater than 10 in both subscales translates into a type D personality. The DS14 has been translated into different languages and has demonstrated high reliability and internal consistency. The Spanish version of DS14 presents adequate psychometric properties with an alpha coefficient of Cronbach values higher than 0.80 on the scale of negative affection and social inhibition.^16^ The DS14 test has shown to have adequate psychometric qualities, to be a stable scale over time, and to have a good internal consistency, independent of mood and health status, as recent studies show.^1^, ^12^, ^17^

Levels of anxiety and depression were assessed with HADS.^18^ The HADS test has also been validated in the Spanish population.^19^ It is made up of fourteen items that are divided into two scales of seven items. Each symptom is scored from 0 to 3 on a 4-point Likert scale according to its strength. Scores higher than 7 on the subscales indicate signs of anxiety or depression; when the marks exceed 10 they translate into a clinical problem. The prevalence of anxiety and/or depression is deduced from scores greater than 7.

### Data analysis

Descriptive statistics were used to explore the characteristics of the patients. Continuous data were expressed as mean and Standard Deviation (SD). For qualitative variables, absolute and relative frequency distributions were estimated. To compare quantitative data between psoriatic patients with and without type D personality the Mann-Whitney *U* test was performed. The Chi-Square test or Fisher's exact test was used for qualitative variables when necessary. Significance was set at p < 0.05. The main outcomes of interest were type D personality and the presence of anxiety and depression. Type D personality was evaluated in a binary way as to present: DS14 score greater than 10 in both subscales; or absent: scores lower than 10. In the same way, HADS is a binary scale with which the prevalence of anxiety and/or depression was considered for scores higher than 7 in each subscale.

## Results

### Baseline characteristics of the sample

A total of 154 patients with moderate to severe psoriasis were invited to join the study. Twenty-one patients did not meet the inclusion criteria and were excluded. Three questionnaires were excluded because they were incomplete. Therefore, 130 patients joined the study. At week 208, there were 24 (18.5%) non-responders: two patients had died and twenty-two did not want to participate. Forty psoriasis patients with type D personality (tDp) and sixty-six patients with psoriasis without tDp were included for analysis. Table 1 summarizes the baseline characteristics of the population at week 0.

**Table 1:** Baseline characteristics of the population week 0.

	TDp (n = 40)	No tDp (n = 66)	p-values
**Age (years)**	52.30 ± 1.97	51.46 ± 1.53	0.74
**Male:Female ratio**	21:19	10:13	0.41
**BMI**	28.50 ± 0.95	28.6 ± 0.74	0.92
**Educational level (%)**			0.15
Primary incomplete	7 (17.50)	4 (6.06)	
Secondary	21 (52.50)	36 (54.55)	
Academic	12 (30)	26 (39.39)	
**Marital status (%)**			0.66
Single	5 (12.50)	12 (18.18)	
Married	30 (75)	48 (71.21)	
Others	5 (12.50)	6 (10.61)	
**Arthropathy (%)**			0.69
Yes	13 (32.50)	19 (28.79)	
**Years of evolution**	22.20 ± 1.93	24.14 ± 1.44	0.41
**Psoriasis treatment (%)**			0.68
Topical	7 (17.50)	9 (13.64)	
Classical systemic drugs	14 (35.00)	20 (30.30)	
Biological drugs	19 (47.50)	37 (56.06)	
**Baseline PASI**	4.48 ± 0.79	4.14 ± 0.62	0.74
**Baseline HADS-A >8**	25 (62.5)	20 (30.30)	0.001
**Baseline HADS-D >8**	19 (47.50)	13 (19.70)	0.002

TDp, type D personality consists of a tendency to inhibit the expression of emotions or behavior to avoid negative reactions from others (Social Inhibition: SI), in combination with the stable tendency to experience Negative Affectivity (NA).

Data are expressed as mean ± standard deviation and as number (percentage); BMI, Body Mass Index; PASI, Psoriasis Area and Severity Index; HADS, the Hospital Anxiety and Depression Scale-Anxiety (HADS-A) and -Depression (HADS-D); p-values refer to the comparison of the patient with or without type D personality.

### Type D personality stability

The prevalence of tDp for participants was 37.7% (95% CI 29.08%–47.23%) at baseline and 27.3% (95% CI 19.77%–36.52%) at week 208. 47.5% (95% CI 32.9%–62.05%) of patients maintained tDp. The stability of tDp was higher in patients with incomplete education vs. basic-higher education and patients with separated-divorced-widowed marital status vs. single-married (Table 2). Higher PASI at week 208 and higher levels of negative affectivity at week 0, beta 0.14 (0.07), p = 0,04 also correlated with greater stability of tDp. No association was observed with age, sex, time of evolution, BMI, treatment, or the absolute reduction of PASI from week 0.

**Table 2:** Type D personality stability at 208 weeks of follow-up.

	tDp Stable (n = 19)	tDp Loss (n = 21)	p-value
**Age (years)**	52.78 ± 2.5	51.8 ± 2.38	0.78
**Male: female ratio**	9:10	12:9	0.53
**BMI**	28.22 ± 1.62	28.75 ± 1.54	0.81
**Educational level primary incomplete vs. secondary-academic**	6 (31.58) vs. 13 (68.42)	1 (4.76) vs. 20 (95.24)	0.03
**Marital status single-married vs. divorced-windowed**	14 (73.68) vs. 5 (26.32)	21 (100.00) vs. 0 (0.00)	0.02
**PASI** (baseline)	5.14 ± 1.08	3.88 ± 1.02	0.40
**PASI** (week 208)	3.67 ± 0.79	1.47 ± 0.76	0.05
**HADS-A >8 (%)**	13 (68.42)	12 (57.14)	0.46
**HADS-D >8 (%)**	11 (57.89)	8 (38.10)	0.20
**Baseline psoriasis treatment (%)**			0.29
Topical	3 (15.79)	4 (19.05)	
Classical systemic drugs	9 (47.37)	5 (23.81)	
Biological drugs	7 (36.84)	12 (57.14)	
**Final psoriasis treatment (%)**			0.39
Topical	1 (5.26)	4 (19.05)	
Classical systemic drugs	2 (10.53)	2 (9.52)	
Biological drugs	16 (84.21)	15 (71.43)	

TDp, type D personality consists of a tendency to inhibit the expression of emotions or behavior to avoid negative reactions from others (social inhibition: SI), in combination with the stable tendency to experience negative affectivity (NA). Data are expressed as mean ± standard deviation and as number (percentage); BMI, Body Mass Index; PASI, Psoriasis Area and Severity Index; HADS, the Hospital Anxiety and Depression Scale-Anxiety (HADS-A) and Depression (HADS-D); p-values refer to the comparison of patients in whom type D personality remains stable and in those who lose it.

### Incidence of type D personality

Table 3 summarizes the type D personality incidence data throughout the 208 weeks of follow-up. During follow-up, 15.15% (10/66) of patients who did not have tDp at week 0 developed tDp. Male sex, the presence of previous depression, anxiety, and high levels of AN at baseline 0.17 (0.06), p = 0.004 increase the risk of developing tDp. In the present study’s sample, at week 208, eighteen of the seventy-seven patients without type D personality (23.4%) scored higher than 10 on the NA subscale.

**Table 3:** Type D personality incidence data throughout the 208 weeks of follow-up.

	Generate tDp (n = 10)	No tDp (n = 56)	p-values
**Age (years)**	51.20 ± 4.2	51.51 ± 1.8	0.95
**Male:Female ratio**	1:4	19:9	0.004
**BMI**	27.63 ± 1.69	28.79 ± 0.71	0.51
**Educational level (%)**			0.37
Primary incomplete	1 (10.0)	3 (5.36)	
Secondary	7 (70.0)	29 (51.79)	
Academic	2 (20.0)	24 (42.86)	
**Marital status (%)**			0.97
Single	2 (20.0)	10 (17.86)	
Married	7 (70.0)	41 (73.21)	
Divorced	1 (10.0)	5 (8.93)	
**PASI** (baseline)	4.04 ± 1.68	4.16 ± 0.71	0.94
**PASI** (week 208)	1.00 ± 0.72	1.63 ± 0.30	0.42
**Baseline HADS-A >8**	6 (60.0)	14 (25.0)	0.03
**Baseline HADS-D >8**	5 (50)	8 (14.29)	0.01
**Baseline psoriasis treatment (%)**			0.92
Topical	1 (10.0)	8 (14.29)	
Classical systemic drugs	3 (30.0)	17 (30.36)	
Biological drugs	6 (60.0)	31 (55.36)	
**Final psoriasis treatment (%)**			0.14
Topical	3 (30.0)	5 (9.09)	
Classical systemic drugs	0 (0.0)	4 (7.27)	
Biological drugs	7 (70.0)	46 (83.64)	

TDp, type D personality consists of a tendency to inhibit the expression of emotions or behavior to avoid negative reactions from others (Social Inhibition: SI), in combination with the stable tendency to experience Negative Affectivity (NA). Data are expressed as mean ± standard deviation and as number (percentage); BMI, Body Mass Index; PASI, Psoriasis Area and Severity Index; HADS, the Hospital Anxiety and Depression Scale-Anxiety (HADS-A) and Depression (HADS-D); p-values refer to the comparison of patients who develop type D and those who do not.

### Psychological implications of type D personality

The incidence of anxiety in patients with tDp was 33.3% (5/15) vs. 10.87% (5/46) in patients without tDp, p = 0.413. There were no differences regarding depression 9.52% (2/21) vs. 5.66% (3/53), p = 0.55. Absolute frequencies of anxiety and depression were higher in patients with tDp at baseline and week 208. The prevalence of anxiety and depression regarding tDp status is shown in Table 4.

**Table 4:** Anxiety and depression prevalence at week 0 and 208.

	Week 0		p-values	Week 208		p-values
	tDp (n = 40)	No tDp (n = 66)		TDp (n = 29)	No tDp (n = 77)	
**HADS-A >8**	25 (62.50)	20 (30.30)	0.001	16 (55.17)	19 (24.68)	0.035
**HADS-D >8**	19 (47.50)	13 (19.70)	0.002	9 (31.03)	7 (9.10)	0.076

TDp, type D personality consists of a tendency to inhibit the expression of emotions or behavior to avoid negative reactions from others (Social Inhibition: SI), in combination with the stable tendency to experience Negative Affectivity (NA).

Data are expressed as number (percentage); HADS, the Hospital Anxiety and Depression Scale- Anxiety (HADS-A) and Depression (HADS-D); p-values refer to the comparison of the patient with or without type D personality.

## Discussion

This study examines the stability of type D personality in patients with moderate to severe psoriasis over 208 weeks of follow-up and its relationship with the development of anxiety and depression.

Several studies have shown the stability of type D personality over time. Zoha et al. found temporal stability for the prevalence of Type D in healthy Israeli adults, the prevalence at baseline was 25.3%, which was not statistically different from the prevalence six years later.^20^ A study by Jellesma et al. shows that the prevalence of type D personality remained stable in Dutch schoolchildren over a period of eighteen months.^21^ Ossola et al. similarly showed that the prevalence of type D was stable over twelve months in Italian intensive care coronary patients.^22^

Lim et al. found that in Korean patients the prevalence of type D personality was similar in patients with Cardiovascular Disease (CVD) with respect to patients with arterial hypertension and without CVD and with respect to controls.^23^, ^24^ In accordance with the above, the present study shows stability of type D personality of 47.5% over the four years of follow-up.

The characteristics of a personality are dynamic traits that throughout life can experience periods of change and stability.^8^ The authors still do not know the reason why patients with Type D personality are more vulnerable, but this could be related not only to biological but also behavioral factors. Bratko and Butkovic showed that genetic factors were primarily responsible for the four-year stability of Eysenck's personality traits – extraversion, neuroticism, and psychoticism – in the transition period from adolescence to young adulthood.^25^ While the changes in personality traits during that period were determined by environmental factors. The stability of the Type D personality, therefore, seems to be due in large part to genetic factors, these factors can contribute to the change of a trait, as well as its continuity.^9^ There are also some environmental factors involved: it seems logical that incomplete education makes the individual understand the disease less which favors the persistence of type D personality. In the same way, it can be affected by vital factors such as having lost a partner.

Within the type D personality, the trait most associated with its stability is having high levels of NA. High-NA makes the person experience more feelings of anxiety, dysphoria, and irritability, generates a negative perspective of himself and reality, thus perceiving imminent problems that are not yet real. In the present study’s sample, 23.4% of patients who did not meet type D personality criteria scored higher than 10 on the NA subscale.

Recent research has shown an increased prevalence of type D personality in patients with psoriasis compared to healthy populations.^4^, ^5^, ^6^ In our study, the prevalence of type D personality is high, (27.3%–37.7%) especially considering they are under treatment as patients of a psoriasis unit. The prevalence is also comparable to the cardiac population (13.5%–35%).^26^

Personality consists of a set of traits that reflect consistencies over time in the general affective level and the behavior of individuals.^27^ In the study by Mols et al. it was seen that anxiety levels were higher in cancer patients and type D personality compared to non-type D.^28^ This is consistent with the literature in patients with and without CVD and in the general population. Type D personality and mood are consistently related, favoring higher levels of anxiety and depression in these patients. Type D personality is consistently related to mood, favoring the presence of anxiety and depression.^12^, ^29^, ^30^ As the authors already know, the interrelation of anxiety, stress and cortisol through the hypothalamic-pituitary-adrenal axis are factors that aggravate psoriasis. Subjects with type D personality and in particular its negative affection subcomponent are more likely to experience negative emotions, depression, anxiety, hostility, irritability, lower levels of self-confidence, and more use of resignation or withdrawal coping strategies.^2^ In the present study’s sample, the incidence of anxiety is three times higher in patients with type D personality throughout the follow-up. Moreover, 23.4% of patients who did not meet type D personality criteria scored higher than 10 in the NA subscale.

Type D personality represents a risk factor for poor prognosis and death in cardiac patients.^1^, ^11^ Its prevalence in patients with CVD ranges between 13.5% and 35%.^29^ This vulnerability, although it remains unknown, could be explained due to genetic and behavioral factors.^31^

There is no previous data on the incidence of type D personality in chronic diseases such as psoriasis, where patients may experience changes in the presence or absence of disease over time and only in acute processes.^11^, ^12^, ^29^ The authors consider that an incidence of 15% is high, considering they are patients under treatment in the psoriasis unit. It seems that the most vulnerable subjects for type D personality are male sex, being under-treated, alterations in mood in the form of anxiety and depression, and again it seems that negative affectivity increases the risk of tDp.

Type D personality is a marker of consistent psychological comorbidity over time and therefore patients with psoriasis and type D personality could be candidates for multidisciplinary team care including psychological care and cognitive behavioral therapy. The authors could consider cognitive-behavioral treatment as a promising framework for intervention. According to previous studies, this could reduce the perceived stigmatization in diseases that affect the skin and thus improve both the psychological results and those related to the disease in patients with psoriasis, it would also help to work the feeling of helplessness in these patients, all of this is significantly correlated with the impact of the disease.^32^, ^33^, ^34^ It is necessary to periodically reassess the presence of type D personality in patients with moderate-severe psoriasis, as new cases may develop over time.

The results of this study should be viewed with caution given some methodological limitations: (1) Limited sample size, (2) Psoriasis severity was limited to moderate to severe, and (3) Patients were under treatment for psoriasis.

## Conclusion

In conclusion, the current study provides further evidence for type-D personality being conceptualized as a stable personality construct instead of a trait phenomenon. Type D personality could represent a frequent personality profile in patients with psoriasis which could identify subjects who are more vulnerable to psychological comorbidity and could benefit from a cognitive-behavioral treatment. It is necessary to periodically reassess the presence of type D personality in patients with moderate-severe psoriasis, as new cases may develop over time.

## Financial support

None declared.

## Authors' contributions

Paula Aguayo-Carreras: Concepts; design; definition of intellectual content; literature search; clinical studies; data acquisition; data analysis; statistical analysis; manuscript preparation; manuscript editing; manuscript review; guarantor.

José Carlos Ruiz-Carrascosa: Concepts; Definition of intellectual content; clinical studies; data acquisition; statistical analysis; manuscript preparation; manuscript editing; manuscript review; guarantor.

Ricardo Ruiz-Villaverde: Concepts; Definition of intellectual content; clinical studies; data acquisition; statistical analysis; manuscript preparation; manuscript editing; manuscript review; guarantor.

Alejandro Molina-Leyva: Concepts; design; definition of intellectual content; clinical studies; data acquisition; data analysis; statistical analysis; manuscript preparation; manuscript editing; manuscript review; guarantor.

## Conflicts of interest

None declared.
